# An effective ensemble learning approach for classification of glioma grades based on novel MRI features

**DOI:** 10.1038/s41598-024-61444-1

**Published:** 2024-05-25

**Authors:** Mohammed Falih Hassan, Ahmed Naser Al-Zurfi, Mohammed Hamzah Alsalihi, Khandakar Ahmed

**Affiliations:** 1 Faculty of Engineering, University of Kufa, Najaf, Iraq; 2 VIPBG, Virginia Commonwealth University, Richmond, VA 23284-3090 USA; 3 Department of Computer Science, Faculty of Computer Science and Information Technology, University of Al-Qadisiyah, Al Diwaniyah, Iraq; 4 Intelligent Technology Innovation Laboratory, Victoria University, Melbourne, VIC 3011 Australia

**Keywords:** Brain tumors, Tumor classification, Ensemble learning, Machine learning, Novel MRI features, Image processing, Machine learning

## Abstract

The preoperative diagnosis of brain tumors is important for therapeutic planning as it contributes to the tumors’ prognosis. In the last few years, the development in the field of artificial intelligence and machine learning has contributed greatly to the medical area, especially the diagnosis of the grades of brain tumors through radiological images and magnetic resonance images. Due to the complexity of tumor descriptors in medical images, assessing the accurate grade of glioma is a major challenge for physicians. We have proposed a new classification system for glioma grading by integrating novel MRI features with an ensemble learning method, called Ensemble Learning based on Adaptive Power Mean Combiner (EL-APMC). We evaluate and compare the performance of the EL-APMC algorithm with twenty-one classifier models that represent state-of-the-art machine learning algorithms. Results show that the EL-APMC algorithm achieved the best performance in terms of classification accuracy (88.73%) and F1-score (93.12%) over the MRI Brain Tumor dataset called BRATS2015. In addition, we showed that the differences in classification results among twenty-two classifier models have statistical significance. We believe that the EL-APMC algorithm is an effective method for the classification in case of small-size datasets, which are common cases in medical fields. The proposed method provides an effective system for the classification of glioma with high reliability and accurate clinical findings.

## Introduction

Machine learning models have made significant achievements in various medical fields such as the classification of Alzheimer’s Disease^1^, COVID-19 Recognition^2,3^, and others^4^. They assist in tasks like image segmentation, tumor detection, and anomaly identification in medical images like X-rays, MRIs, and CT scans^5^. One of the most life-threatening types of tumors is a malignant brain tumor which is associated with a high mortality rate^6^. Cancer Research UK (CRUK) states that in the last forty years, the rates of malignancy have increased by about 39% in the UK. Brain tumors can grow rapidly, become more aggressive, and eventually lead to death. There are several types of brain tumors and one of the most common types is glioma, which can be classified into four grades (I, II, III, IV) according to the World Health Organization (WHO). Low-grade gliomas, which include grade I and grade II, grow very slowly with a significantly better prognosis^7^. High-grade gliomas, which include grades III and IV, are managed with primary chemotherapy, radiotherapy, or resection. It is very important to distinguish between low-grade and high-grade gliomas before surgical intervention because this effectively affects the treatment approach and the patient’s health during the recovery phase^8^.

Malignant brain tumors such as glioma can be diagnosed based on the traditional way, which is based on the visual assessment of the various attributes of MRI medical images. However, making a good decision needs a high level of experience in the neuroradiology field. Furthermore, the inconsistency and heterogeneity of many visual characteristics of malignant brain tumors lead to very complicated issues in the diagnosis^7,8^. For all those reasons, this study aims to design and develop a classification model of malignant grades glioma that can help the specialist achieve accurate classification of glioma grades with a minimum error rate. In other words, developing a classification model for glioma grades using a statistical analysis of tumor descriptors, led to achieving an accurate differentiation between glioma grades, which assists physicians in distinguishing them.

Many factors and MRI characteristics can be used in the clinical center for brain tumor diagnosis. For example, the analysis of necrosis, edema, enhancement of non-enhanced MRI tumors and different MRI features that appear after tumor enhancement^6^. Furthermore, determining the malignancy grade of glioma depends on the specialist’s experience and level of qualifications. The diagnosis of an MRI brain tumor on visual examination through magnetic resonance image analysis may take a long time as it requires strong experience for the result of the diagnosis to be accurate^9^. In addition, with the enhancement of the MRI protocols and the development in this industry, the diagnosis of glioma grading based on visual diagnosis is considered difficult^10^. Therefore, in attempting to enhance the sensitivity and quality of classification methods, we proposed novel MRI features integrated with an effective ensemble learning method.

Using machine learning in the detection and classification of malignant brain tumors poses’ several challenges^4,5^. For example, limited data availability of brain tumor images can affect the performance and generalization ability of models. In addition, machine learning models are prone to overfitting, especially when dealing with limited data. Data imbalance is another challenge in which the distribution of different types of brain tumors in datasets can be highly imbalanced. This can lead to biased models and difficulty in accurately detecting malignant tumors. Brain tumors exhibit considerable heterogeneity in terms of size, shape, texture, and location. The selected feature extraction method and machine learning models need to be robust enough to accurately classify tumors despite these variations. Addressing these challenges requires careful design that takes the consideration all the challenges previously mentioned.

To address these challenges, we proposed an automated classification model based on a novel feature extraction method integrated with an effective machine-learning algorithm called EL-APMC^11^. EL-APMC is built on an ensemble of base classifiers that adaptively combine to maximize classification results. This structure allows for several benefits; for example, incorporating more classifiers can effectively reduce overfitting and improve their generalization performance on unseen data. In addition, EL-APMC is trained using bootstrap bagging without replacement which can mitigate the effects of class imbalance. Unlike other ensemble learning methods that use fixed fusion methods, EL-APMC uses an adaptive fusion method called Power Mean Combiner (PMC) that is trained to match data statistics which results in maximizing classification accuracy. Also, we used the subspace training method to maximize independence among base classifiers and improve diversity which brings benefits such as improved accuracy, robustness, and reduced overfitting. As a result, the EL-APMC algorithm is considered a promising technique that is used to classify small-size datasets which is a common problem in the medical domain. The effectiveness EL-APMC algorithm is compared with twenty-one machine learning methods which are considered state-of-the-art machine learning. The findings indicate that the EL-APMC algorithm demonstrated notable performance in both classification accuracy (88.73%) and F1-score (93.12%) when evaluated on the BRATS2015 MRI Brain Tumor dataset. In addition, this work investigates the effectiveness of the proposed MRI features on the classification of glioma grades.

The rest of the paper is organized as follows. The recent literature related to the brain tumors classification is introduced in Section II. Section III discusses the impact of dataset size on classification performance. Section IV reviews the feature extraction method and working principles of EL-APMC algorithms. Section V discusses the paper’s results. Finally, section IV gives the main conclusion and future direction.

## Related work

Various machine learning methods have been used and proposed in recent years to classify brain tumors as shown in Table 1. In the last few years, the use of machine learning and the application of AI increased rapidly and many researchers have proposed different classification methods. In^12^, MRI glioma grades have been classified into three grades (II, III, and IV). The classification system was developed using Gabor texture as input features and SVM was selected as the classification model. The results show a classification accuracy of 88%. While^13^, has proposed a classification system based on statistical MRI features and K-means clustering to differentiate low grades from high grades of MRI brain tumors and achieved a classification accuracy of 80.40%. Similarly, MRI images have been classified into two classes (normal and abnormal)^14^. The proposed model consists of many phases starting with an enhancement of the brain MRI images using Shift-Invariant Shearlet Transform (SIST). Then researchers proposed the Gabor Grey Level Co-occurrence Matrix (GLCM) and Discrete Wavelet Transform (DWT) for the features extraction phase. Finally, these selected features were fed to a feed-forward backpropagation neural network and obtained an accuracy rate of 99.8%. Hsieh et al.^15^ suggested a classification model using logistic regression to classify low grades against high grades based on Local Binary Pattern (LBP) texture features and achieved a classification accuracy of 93%. Deep learning based on CNN has also been proposed to classify MRI glioma grades^16^. The work has accomplished a classification accuracy of 91.16%. Shree et al.^17^ proposed a brain tumor classification model for binary classification (normal and abnormal). They used GLCM for feature extraction and a PNN classifier, which resulted in 95% classification accuracy. The mean intensities of the MR regions were used to produce a classification system for glioma grades using SVM as a classification method^18^ and the obtained classification result was 93%. Likewise^19^, proposed an automatic tumor detection and segmentation based on a hybrid energy-efficient method for automatic tumor detection and segmentation. The developed methods consist of seven long phases to achieve 98% accuracy. In^20^, a two-stage ensemble learning approach is proposed to classify three glioma grades (Glioma Grade II, Glioma Grade III, and Glioma Grade-IV). The number of subjects used in the study is 135 (90 patients and 45 controls) and five characteristics are used in classification which is human telomerase reverse transcriptase (hTERT), chitinase-like protein (YKL-40), interleukin 6 (IL-6), tissue inhibitor of metalloproteinase-1 (TIMP-1) and neutrophil/lymphocyte ratio (NLR). They claimed to achieve better classification accuracy compared to the state-of-the-art machine learning classifiers. The work given in^21^ used anisotropic noise removal filtering, GLCM for feature selection, and SVM classifier to identify the tumor region from brain MRI images. According to their results, they can localize tumor regions with 98% accuracy. Rajeev et al.^22^ investigated a hybrid deep learning approach for brain tumor classification, by using an improved Gabor wavelet transform and BiLSTM network. The experiments have been done based on the Kaggle dataset which is public and open source, the dataset includes four directories such as glioma-tumor, meningioma-tumor, no-tumor, and pituitary-tumor. The proposed methods have been implemented using the MATLAB platform and the highest performance accuracy was achieved at 98.4%. An automated classification system for the segmentation of MRI brain tumors has been accomplished based on the combination of the Interval Type-II fuzzy logic system and an artificial bee colony algorithm to identify tumor regions^23^. The developed algorithm has investigated using image sequences available in the BRATS challenge datasets (2015, 2017, and 2018). The researcher claimed to achieve 96% classification results in terms of the Dice-Overlap Index (DOI). The summary of the classification models and features used for the classification of glioma grades and their details are shown in Table 1.

**Table 1:** Summary of works used various classification models for glioma grading.

Author	Year	Dataset size	Features extraction method	Classification method	Accuracy (%)
Zacharaki et al.	2009	102 brain tumors: II (22), III (18), glioblastomas (34)	Gabor filter texture analysis	SVM	88
Inano et al.	2014	14 (low grade), 19 (high grade)	Statistical MRI-features	K-mean clustering	80.40
Arunachalam	2017	230 MRI images	Gabor, GLCM, and Discrete wavelet transform (DWT)	Feed-forward back propagation neural network	99.8
Hsieh et al.	2017	34 glioblastomas and 73 lower-grade gliomas	Texture-LBP	Logistic regression	93
Khawaldeh et al.	2017	109 subjects	–	CNN	91.16
Shree and Kumar	2018	650 MRI images	Grey level co-occurrence matrix	Probabilistic neural network (PNN)	95
Citak-Er et al.	2018	34 patients I (3), II (12), III (8), IV (20)	Statistical measures from advanced MRI, mean of intensities of the MR regions	SVM	93
Rajan and Sundar	2019	41 MRI images	Adaptive grey-level co-occurrence matrix (AGLCM)	Support vector machine (SVM)	98
Joshi et al.	2021	135 cases (90 patients and 45 controls)	–	Ensemble-based approach (multi-grade classification)	83.33
Rajeev et al.	2022	Kaggle dataset	Gabor wavelet transform	BiLSTM network	98.4
Rasheed et al.	2023	DICOM datasets	Grey-level co-occurrence matrix (GLCM)	SVM	98
Alagarsamy et al.	2023	BRATS datasets (2015, 2017, and 2018)	–	Artificial bee colony and interval type-II fuzzy logic system	DOI = 96

## Impact of dataset size on classification performance

In this section, we review the challenges of training machine learning models on small data sizes and investigate the most effective machine learning algorithms that target this issue. Dataset plays a pivotal role in modern healthcare services for example in personalized medicine and automated diagnosis^24^. The size of data is considered a crucial factor in determining the performance of a machine learning algorithm. In practice, small data size leads to overfitting problems while large data size leads to better classification results^25–27^.

Data collection in the medical area faces many obstacles such as rare medical conditions and medical organizations’ privacy. Deep learning algorithms provide good results in different applications. However, to get an accurate result with a deep learning algorithm it is necessary to train it with a large amount of data which in some cases is not available^28^. In addition, training machine-learning algorithms on large data sizes require a considerable amount of time and computation resources that may not be available in certain circumstances.

Many efforts in literature tried to define the size of small datasets but there is no clear definition for that. For example, Shawe-Taylor et al.^29^ presented a method that specifies the minimum number of features to achieve the desired accuracy called Probably Approximately Correct (PAC). While^30^ proposed, an algorithm based on information theory for defining a minimum data size. Other work^31^ examined different works that dealt with small data sizes to define a range for small dataset sizes.

Training a machine-learning algorithm on a small data size is a challenging task since the data does not represent the actual data distribution, which may lead to an overfitting problem. In an overfitting situation, the classification algorithm performs well on training data and provides poor performance on testing data. In other words, the fitted algorithm is generalized well on training data which does not represent the actual data distributions. In this case, the trained model is not generalized well and leads to unreliable and biased classification results. Increasing the accuracy of classification on limited data size is a challenging research area. To address this problem, some literature focused on increasing the accuracy of the classification algorithm on a limited-size dataset while others investigated the effect of the dataset size on the performance of the classification algorithm^32,33^. In this work, EL-APMC as well as the state-of-the-art machine learning methods are investigated to tackle the problem of classification limited data size. In the following sections, we reviewed our proposal and compared its performance against the state of art machine learning algorithms. Then, the comparison is evaluated among different classification metrics.

## Proposed method

In this section, we reviewed several MRI descriptors of brain tumors that are used to extract eight novel features. Then we described the structure of the EL-APMC algorithm that used to develop an automated classification system for glioma grades.

### Feature extraction

**Figure 1:**
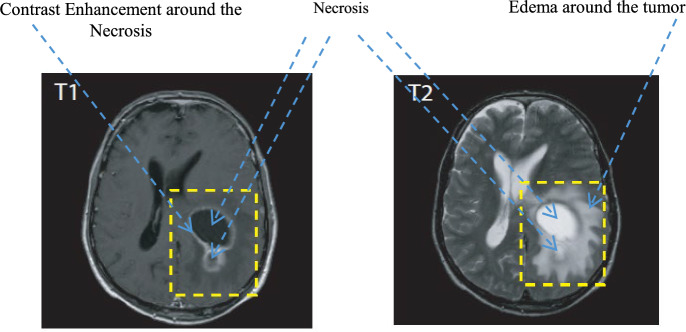
The MRI images of Grade IV glioma exhibit distinct characteristics in terms of the morphology of the brain tumor. These characteristics include the presence of tumor enhancement in the T1 images after contrast enhancement. The center of the tumor is marked by necrosis, while edema surrounds the tumor and is visible in the T2 images.

In this experimental work, standard labeled datasets were used to evaluate the proposed approach, namely BRATS2015^34^. This dataset has a labeled identification layer and it is used to generate four masks to individually bring in labeled regions. These regions include necrosis, edema, non-enhanced, and enhanced tumors. Visualizations of these brain tumor descriptors show different recognized regions for a brain tumor, which are extracted using T1 with enhancement as shown in Fig. 1.

The presence of tumor descriptors is measured by utilizing the number of pixels within each labeled region of the tumor. A search process is conducted to determine the total number of pixels in each region across all slices. This procedure is carried out for all patients in the dataset. Subsequently, an average of the results is calculated for each patient. The following equation is used to determine four MRI features that are used in this work.

1 $$Name\_M = \frac{1}{z}\mathop \sum \limits_{i = 1}^{x} \mathop \sum \limits_{j = 1}^{y} \mathop \sum \limits_{k = 1}^{z} \left\{ {\begin{array}{*{20}l} {1\;if\;SEG\left( {x,y,z} \right) = Descriptor\;label} \hfill \\ {o\;othwise} \hfill \\ \end{array} } \right.$$

The average presence of tumor descriptors denoted as $Name\_M$, is calculated based on the label identification layer (*SEG*) provided by the dataset. z represents the total number of MRI slices that contain a tumor, while x and y represent the coordinates of each MRI slice. An additional four novel features are extracted and involved in the classification process. These features are measured based on the following equations;

2 $$tC\_R = \frac{tC\_M}{{tC_{M} + Nec_{M} + Edm\_M + tnC\_M}}$$

3 $$tnC\_R = \frac{tnC\_M}{{tnC_{M} + Nec_{M} + Edm\_M + tnC\_M}}$$

4 $$Edm\_R = \frac{Edm\_M}{{tnC_{M} + Nec_{M} + Edm\_M + tnC\_M}}$$

5 $$Nec\_R = \frac{Nec\_M}{{tnC_{M} + Nec_{M} + Edm\_M + tnC\_M}}$$

$Names\_M$ takes the following values $tC\_M$, $tnC\_M$, $Edm\_M$, and $Nec\_M$. Where $tC\_M$, $tnC\_M$, $Edm\_M$, and $Nec\_M$ are the average presence of contrast enhancement, non-enhancement, edema, and necrosis respectively. They are calculated from (1) where $tC\_R$, $tnC\_R$, $Edm\_R$, and $Nec\_R$ are the resultant ratios of tumor enhancement, non-enhancement, edema, and necrosis respectively.

### Ensemble learning based on adaptive power mean combiner (EL-APMC)

EL-APMC is a classification method proposed in^11^, which belongs to the family of ensemble learning methods. In this work, a theoretical framework is developed to understand how the fusion methods for ensemble learning systems interact with base classifiers. Based on the theoretical results a new adaptive classification method is proposed and achieved notable results against several fusion methods. In this work, we investigate the strengthening of the classification accuracy of the EL-APMC algorithm and compare it with the state-of-the-art machine learning algorithms in case of limited dataset size. The fusion method used in the EL-APMC is called power mean combiner (PMC) and is defined as follows

6 $$f_{\alpha } \left( {k_{1} ,k_{2} , \ldots k_{N} } \right) = \left( {\frac{1}{N}\mathop \sum \limits_{i = 1}^{N} k_{i}^{\alpha } } \right)^{1/\alpha } ,\quad where\; - \infty < \alpha < \infty$$

where $k_{1} ,k_{2} , \ldots ,k_{N}$ are positive real numbers that represent base classifiers outputs and $\alpha$ is a real number that represents the aggregation method used in $f_{\alpha } \left( . \right)$. PMC refers to a function that combines infinite arithmetic fusion operations, including arithmetic, geometric mean, harmonic mean, and more. However, it is unclear why certain fusion methods work better than others for a given classification task. Fortunately, PMC can aggregate infinite fusion functions, and we can search for an optimal function that minimizes classification error.

The working principle of the EL-APMC is described as follows. The ensemble setup consists of two main phases: training and testing. During the training phase, a fivefold cross-validation approach is employed. In each fold, the data is pre-processed before being used to train individual classifiers. The goal of the pre-processing stage is to introduce diversity among the base classifiers. To achieve this, we employ two well-known methods, namely bagging, and subspace. The combination of bagging and subspace techniques enhances randomness and minimizes the generalization error at the decision combiner stage. In bagging, a bootstrap method is utilized, it is a technique of generating multiple bootstrap samples from the original training dataset to train individual base learners within the ensemble. Each bootstrap sample is created by randomly sampling observations from the original dataset without replacement, resulting in multiple subsets that may contain duplicate instances. These subsets are then used to train each base learner independently which helps improve the diversity among the base learners. This is crucial for enhancing the overall performance and robustness of the ensemble model. By training base learners on different subsets of the data, bootstrapping reduces the risk of overfitting and helps capture different aspects of the underlying data distribution.

The bootstrap process generates N subsets each generated bootstrap subset is divided into two equal parts: one for In-Bag (InBag) samples and the other for Out-of-Bag (OutBag) samples. The InBag portion is utilized to train the N base classifiers, while the OutBag samples are used to estimate individual classifier weights, which are later used in the decision combination process. Additionally, all the OutBag replicas are aggregated and utilized to train the PMC. This setup offers the advantage of eliminating the need for additional data to train the PMC, as the OutBag samples are used for this purpose. Bagging with bootstrap aggregating is considered a regularization technique that reduces overfitting and improves generalization performance. Another method used to control regularization is early stopping which is a method used to prevent overfitting by halting the training process when the performance on a validation set starts to degrade. In our proposal, we can control the number of base classifiers (N) that are used in the ensemble to prevent them from becoming overly complex.

The second method employed to enhance diversity is the random subspace technique. Instead of using the entire feature set for training each base model, a random subset of features is selected for each model. After selecting the feature subset, each base model is trained on the corresponding subset of features. This results in improving the performance and robustness of ensemble learning, particularly in scenarios where overfitting is a concern or where datasets have high dimensionality. Using bootstrap bagging and random subspace training as well as performing thorough hyperparameter tuning can mitigate underfitting in ensemble learning and improve the predictive performance of the model.

The number of features used is determined by taking the square root ($m_{r}$) of the total number of predictors generated from the bootstrap sampling. In the final stage of training, the aggregated replicas of OutBag samples are employed to train PMC. The approach used to implement PMC is called Adaptive PMC with Threshold Estimation (APMCT). This method involves estimating the probability density functions (pdfs) of the classes with an optimal threshold. An adaptive algorithm is utilized to estimate the prior and posterior probabilities of the combiner. For the two classes case, the optimal threshold is determined by minimizing the classification error using the following formula.

7 $$P_{e} = P\left( {w_{1} } \right)F_{1} \left( {\mu_{opt,} m_{1} ,\sigma_{1} } \right) + P\left( {w_{2} } \right)F_{2} \left( {\mu_{opt,} m_{2} ,\sigma_{2} } \right)$$

where $P_{e}$ is the classification error, $P\left( {w_{j} } \right) ,j = 1,2$ is the classes’ prior probabilities, $F_{j} \left( . \right),j = 1,2$ is the cumulative distribution function of the class $w_{j}$, and $\mu_{opt,}$ is the optimal threshold. $F_{j} \left( . \right)$ is estimated using the histogram technique. During the training phase, the EL-APMC algorithm minimizes $P_{e}$ according to the following formula

8 $$min\left( {P_{e} \left( x \right))} \right. , x \in \left( {\alpha_{opt} ,\mu_{opt} } \right)$$

There are many optimization algorithms available to solve (8), among these, we used surrogate optimization. It refers to a method used in optimization algorithms where a surrogate model is employed to approximate the behavior of a complex, computationally expensive, or difficult-to-evaluate objective function. Instead of directly evaluating the objective function, which might involve time-consuming simulations or expensive experiments, the surrogate model is used as a proxy to guide the optimization process. This involves iteratively updating the surrogate model based on a limited set of evaluations of the true objective function. Then the surrogate model is used to predict the objective function values at unexplored points in the search space. These predictions are used to select new points to evaluate the true objective function, aiming to improve the overall optimization process efficiently. The primary advantage of surrogate optimization is its ability to reduce the computational cost of optimization by replacing expensive function evaluations with inexpensive surrogate model predictions^35^.

**Figure 2:**
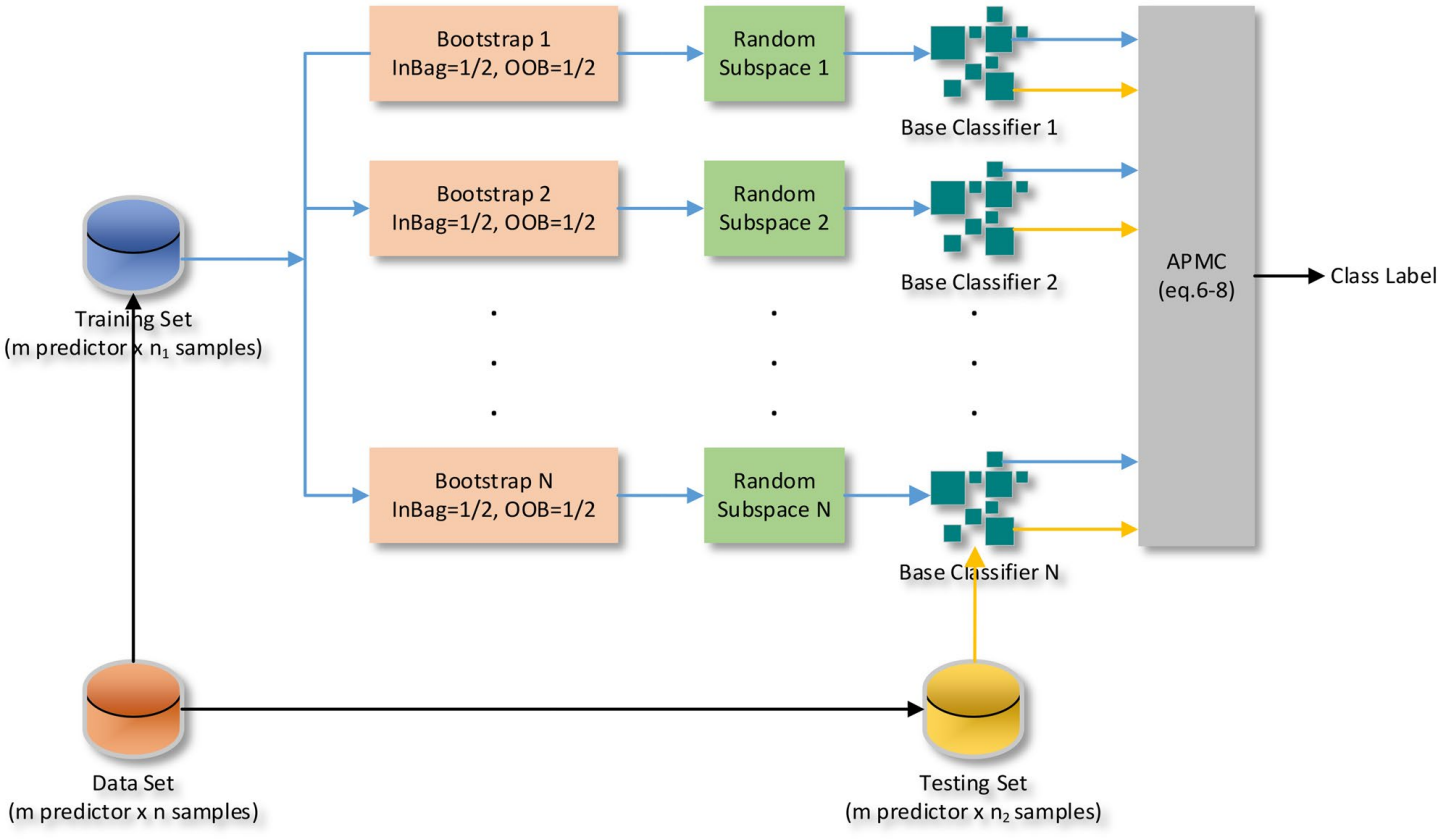
Workflow principles of EL-APMC.

Using (8), $\alpha_{opt}$ and $\mu_{opt}$ are estimated and used to classify data in the test phase. It can summarize the working principle of the EL-APMC in algorithm 1 and Fig. 2 shows the working principles of the EL-APMC Algorithm.

**Algorithm 1:**
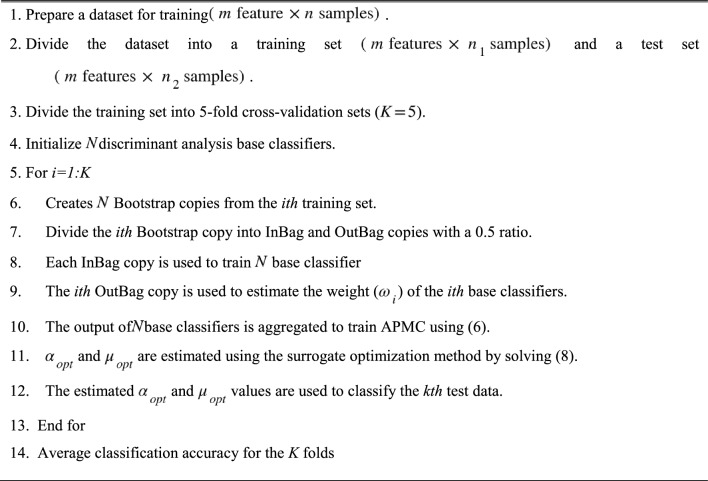
EL-APMC Algorithm.

## Result analysis and discussion

In this section, we investigate the effectiveness of the integration of the proposed MRI features with different machine learning algorithms as well as the EL-APMC algorithm for the classification of glioma grades based on the BRATS 2015 dataset. The environment used for the classification is MATLAB^36^ since it has various tools that support machine learning tasks. We evaluated the glioma grade classification dataset on twenty-one machine learning models available in MATLAB that represent the state-of-the-art machine learning models. As known^37^ deep learning algorithms are only effective in large datasets and fail to achieve a good performance in small datasets size. Since our dataset size is about 275 instances, we have not included deep learning algorithms in the comparison. Table 2 shows the basic default parameter values for the 21 classifier models. The classification results are averaged over fivefold cross-validation. The parameters used in the training EL-APMC algorithm are defined in Table 3.

**Table 2:** Classification models and their parameter values.

No	Classification model	Parameter values
1	Logistic regression	Standard parameters
2	Linear SVM	Kernel function: Linear Kernal scale: Automatic Box constraint level: 1
3	Ensemble bagged trees	Ensemble method: Bag Learner type: Decision tree Maximum number of splits: 350 Number of Learners: 100
4	Ensemble subspace discriminant	Ensemble method: Subspace Learner type: Discriminant Number of Learners: 100 Subspace dimension: 17
5	Linear discriminant	Covariance structure: Full
6	Medium Gaussian SVM	Kernel function: Gaussian Kernel scale: 5.8 Box constraint level: 1
7	Quadratic SVM	Kernel function: Quadratic Kernel scale: Automatic Box constraint level: 1
8	Coarse Gaussian SVM	Kernel function: Gaussian Kernel scale: 23 Box constraint level: 1
9	Weighted KNN	Number of neighbors: 10 Distance metric: Euclidean Distance weight: Squared inverse
10	Gaussian naive Bayes	Distribution name for numeric predictors: Gaussian Distribution name for categorical predictors: MVMN
11	Ensemble subspace KNN	Ensemble method: Subspace Learner type: Nearest neighbors Number of learners: 100 Subspace dimension: 17
12	Medium KNN	Number of neighbors: 10 Distance metric: Euclidean Distance weight: Equal
13	Cubic KNN	Number of neighbors: 10 Distance metric: Minkowski (cubic) Distance weight: Equal
14	Decision tree	Maximum number of splits: 4 Split criterion: Gin’s diversity index Surrogate decision splits: off
15	Kernel naive Bayes	Distribution name for numeric predictors: Kernel Distribution name for categorical predictors: MVMN Kernel type: Gaussian
16	Cubic SVM	Kernel function: Cubic Kernel scale: Automatic Box constraint level: 1
17	Ensemble RUS boosted trees	Ensemble method: RUSBoost Learner type: Decision tree Maximum number of splits: 20 Number of learners: 100 Learning rate: 0.1
18	Fine KNN	Number of neighbors: 1 Distance metric: Euclidian Distance weight: Equal
19	Ensemble boosted trees	Ensemble method: AdaBoost Learner type: Decision tree Maximum number of splits: 20 Number of learners: 100 Learning rate: 0.1
20	Cosine KNN	Number of neighbors: 10 Distance metric: Cosine Distance weight: Equal
21	Fine Gaussian SVM	Kernel function Gaussian Kernel scale: 1.5 Box constraint level: 1

**Table 3:** EL-APMC parameters.

Parameter	Definition	Range
Ensemble learning method	Bootstrap bagging and random Subspace	Defaults setting^36^
Number of features ($Nof$) Selected for Random Subspace ($m_{r}$)	$m_{r} = \sqrt {NoF}$	$1 \le m_{r} \le NoF$
Percentage of InBag samples	0.5	$0 < {\text{InBag }} < 1;$ ${\mathrm{InBag}} = 1 - {\mathrm{OutBag}}$
Percentage of OutBag samples	0.5	$0 < {\text{OutBag }} < 1;$ ${\mathrm{OutBag}} = 1 - {\mathrm{InBag}}$
Base classifier	Linear Discriminant	Defaults setting^36^
Number of base classifiers	$N = 100$	$1 \le {\mathrm{N}} < \infty$
Power mean combiner	$f_{\alpha } \left( {k_{1} ,k_{2} , \ldots k_{N} } \right) = \left( {\frac{1}{N}\mathop \sum \limits_{i = 1}^{N} k_{i}^{\alpha } } \right)^{1/\alpha }$	$- \infty < \alpha < \infty$
Optimization method	Surrogate optimization	Defaults setting^36^

Many evaluation metrics have been measured and evaluated such as the classification accuracy, recall, precision, and F1 score. These metrics are the most familiar tools used to measure the performance of a classification model. All these metrics measures are derived from the confusion matrix defined in Table 4. Where True Positive (TP) represents the number of instances that the model predicts as positive where they are actual positive instances. False Negative (FN) is the number of instances that the model predicts as negative where they are actual positive instances. False Positive (FP) is the number of instances that the model predicts as positive where they are actual negative instances. True Negative (TN) is the number of instances that the model predicts as negative where they are actual negative instances. The performance measures metrics such as accuracy, recall, precision, and F1-score are derived from confusion matrix parameters (TP, FN, FP, and TN) as defined in (9)–(12). The accuracy metric measures the ability of a model to identify the true total positive and negative instances compared to the total instances. In the case of the imbalanced dataset, the accuracy measure provides inaccurate results since the class with a high majority overwhelms the minority class. The recall metric tries to capture how many positive instances are predicted compared to the actual positive instance. This will be beneficial in case there is a high cost related to the prediction of false negatives. Precision metric measures how accurate the classification model is in predicting positive instances, in other words, how many of them are actual positive instances. This will be beneficial in case there is a high cost related to the false positive. The F1-score metric is the harmonic mean of the recall and precision metrics, it will benefit when both recall and precision are important and the average results of both metrics are needed.

9 $$Accuracy = \frac{TP + TN}{{TP + TN + FP + FN}}$$

10 $$Recall = \frac{TP}{{TP + FN}}$$

11 $$Precision = \frac{TP}{{TP + FP}}$$

12 $$F_{1} = \frac{2}{{recall^{ - 1} + precision^{ - 1} }} = \frac{2TP}{{2TP + FP + FN}}$$

**Table 4:** Details of confusion matrix.

	Predicted condition	
Actual Condition	True positive (TP)	False negative (FN)
	False positive (FP)	True negative (TN)

The classification results are evaluated against four metrics, which are accuracy, recall, precision, and F1-score over 22 classifier models. Table 5 shows the performance of classification models ranked in terms of classification accuracy in decreasing order.

**Table 5:** Performance of classifier models in terms of accuracy, recall, precision, and F1-score.

Classifier		Accuracy (%)	Recall (%)	Precision (%)	F1-Score (%)
1	EL-APMC	88.73	97.59	89.05	93.12
2	Logistic regression	87.59	95.00	90.09	92.48
3	Linear SVM	87.59	97.27	88.43	92.64
4	Ensemble bagged trees	87.59	94.09	90.79	92.41
5	Ensemble subspace discriminant	87.59	96.82	88.75	92.61
6	Linear discriminant	86.86	96.36	88.33	92.17
7	Medium Gaussian SVM	86.86	97.27	87.70	92.24
8	Quadratic SVM	86.50	96.36	87.97	91.97
9	Coarse Gaussian SVM	86.50	98.64	86.45	92.14
10	Weighted KNN	86.50	96.36	87.97	91.97
11	Gaussian naive Bayes	86.13	92.73	90.27	91.48
12	Ensemble subspace KNN	86.13	97.27	86.99	91.85
13	Medium KNN	85.40	96.36	86.89	91.38
14	Cubic KNN	85.04	97.27	85.94	91.26
15	Decision tree	84.67	88.64	91.98	90.28
16	Kernel naive Bayes	84.67	89.55	91.20	90.37
17	Cubic SVM	84.31	90.91	89.69	90.29
18	Ensemble RUS boosted trees	84.31	85.91	94.03	89.79
19	Fine KNN	83.94	91.82	88.60	90.18
20	Ensemble boosted trees	83.21	93.64	86.55	89.96
21	Cosine KNN	82.12	91.82	86.70	89.18
22	Fine Gaussian SVM	80.29	99.09	80.74	88.98

**Figure 3:**
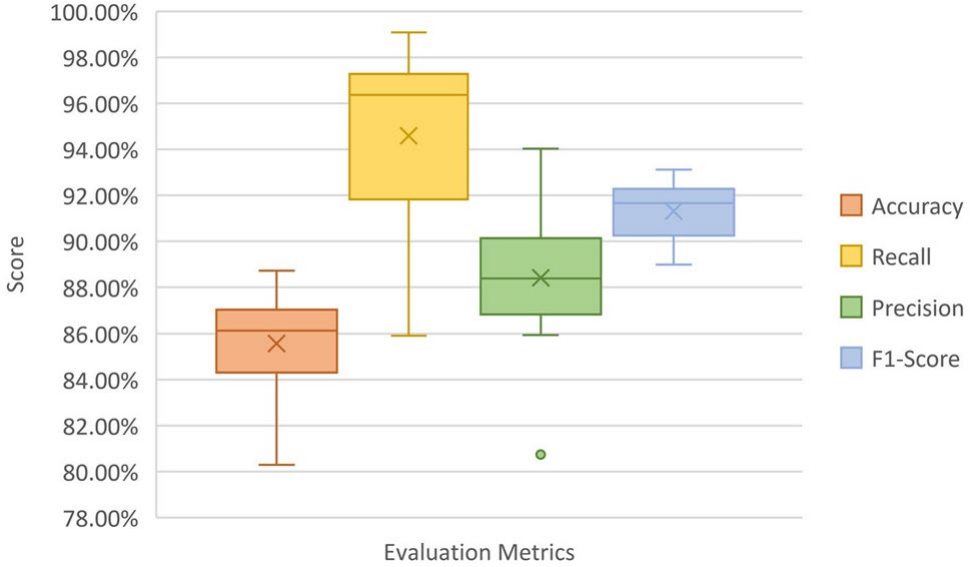
Box plot for different evaluation metrics.

Figure 3 shows a comparison among different evaluation metrics using the box plot. The purpose of the comparison is to statistically summarize the performance of classifier models among evaluation matrices. As shown classifiers show large variability across recall scores compared to other metrics, while the variability is minimal regarding the F1-score. This is because the F1-score takes the harmonic mean of recall and precision resulting in reducing the variability. The average score results among different metrics are 85.57%, 94.58%, 88.38%, and 91.31%, for accuracy, recall, precision, and F1-score respectively.

**Figure 4:**
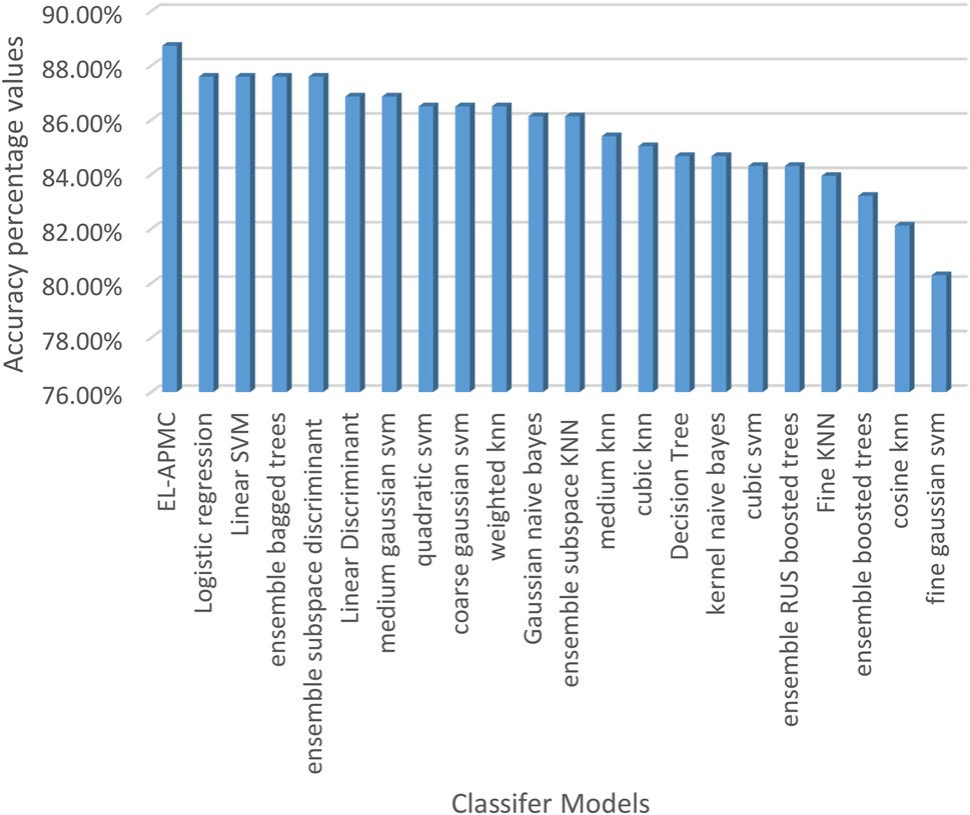
Ranking classifiers models according to their classification accuracy values.

**Figure 5:**
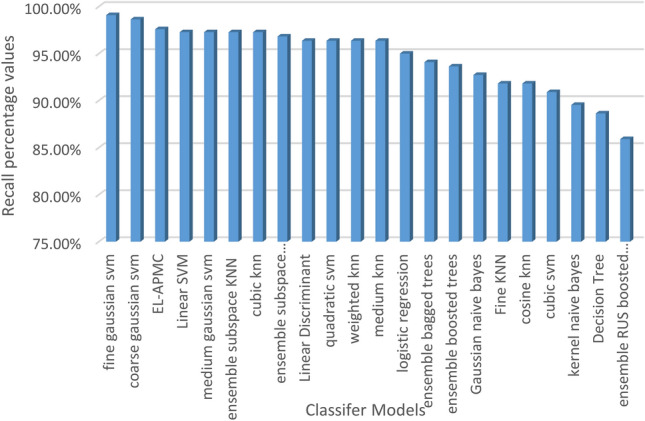
Ranking classifiers models according to their recall values.

**Figure 6:**
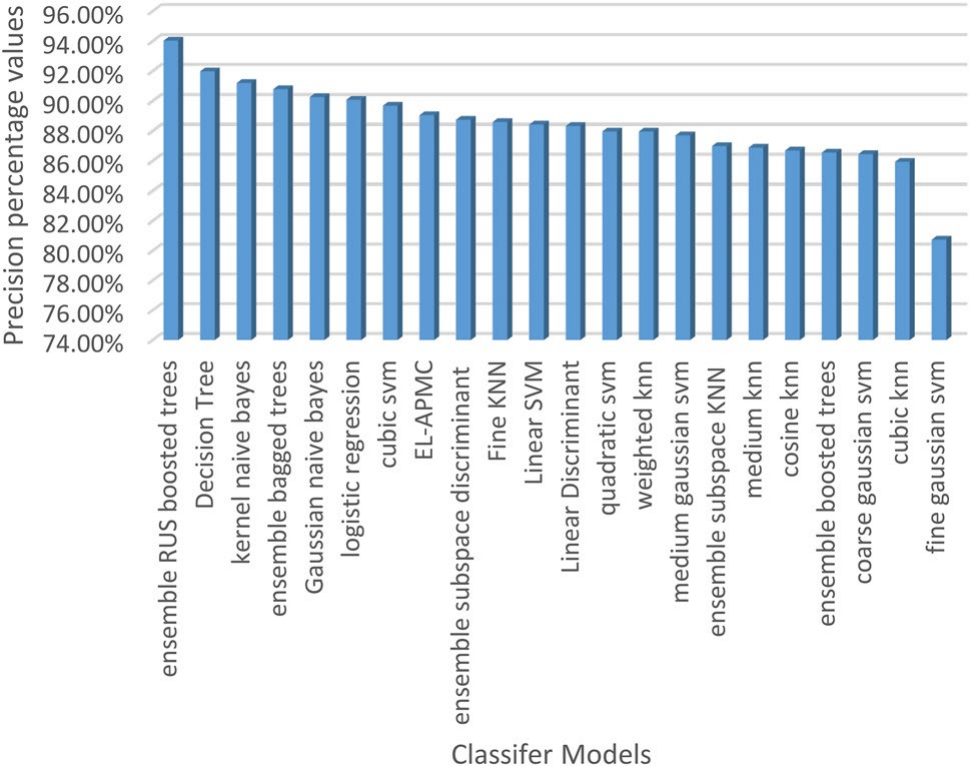
Ranking classifiers models according to their precision values.

**Figure 7:**
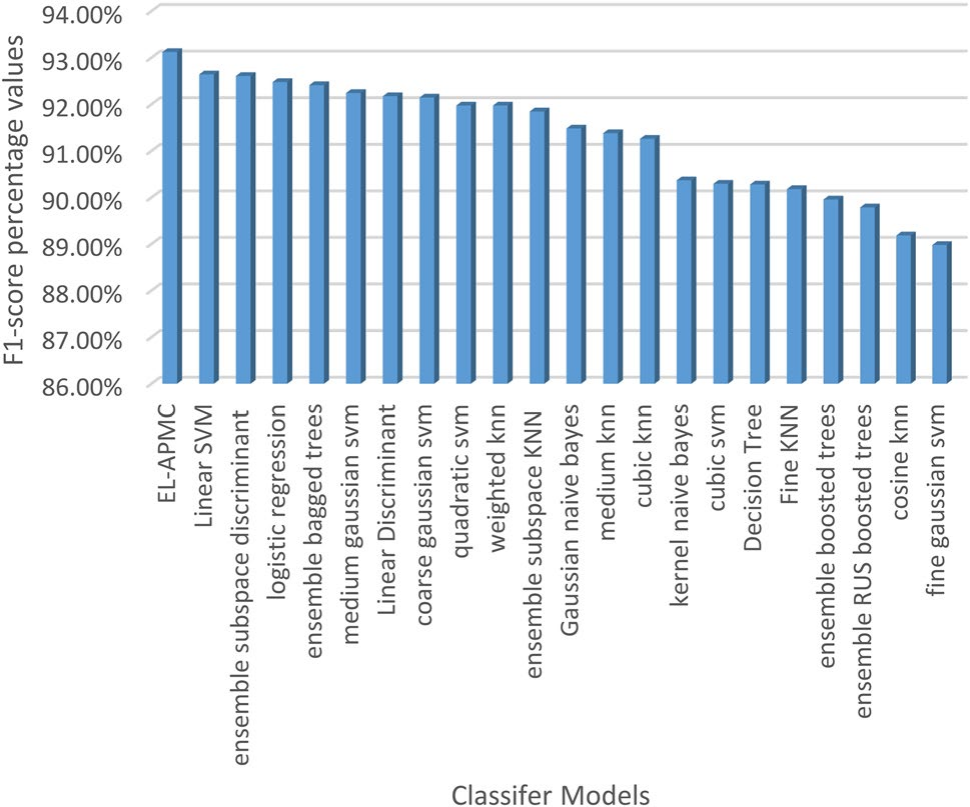
Ranking classifiers models according to their F1-score values.

Figures 4, 5, 6 and 7 visualize the results of Table 5 in terms of accuracy, recall, precision, and F1-score. As shown in Fig. 4, the EL-APMC algorithm achieved the best performance in terms of classification accuracy compared to the 21 Classifier models under comparison and logistic regression scored second and linear SVM scored third. Figure 5 shows the performance of classifiers ranked in descending order in terms of recall metric where fine Gaussian SVM ranks first followed by coarse Gaussian SVM ranks second and the EL-APMC algorithm ranks third. It is obvious from the previous results that the variants of SVM classifiers work best for precision metric. Figure 6 shows the classifier models rank in descending order in terms of precision metric where ensemble RUS boosted trees rank first followed by decision tree ranks second, Kernel Naive Bayes ranks third and the EL-APMC algorithm ranks 8th place. It is clear from the previous results that the variants of decision tree classifiers work best for precision metric.

The F1 score measures the average values of recall and precision and it is considered a crucial metric in the case of an imbalanced dataset where the accuracy metric provides inaccurate results. Figure 7 shows the performance of classifier models in terms of F1-score ranked in descending order. The EL-APMC algorithm ranks in first place followed by Linear SVM which ranks second and ensemble subspace discriminant ranks third place. In summary, the EL-APMC algorithm provides the best performance in terms of accuracy and F1 score since its structure combines two strategies that help in achieving these results.

First, the EL-APMC model uses the idea of ensemble learning in which instead of using a single classifier model, the EL-APMC model uses an ensemble of machine learning models that improves the classification performance. Second, unlike popular ensemble learning methods that use the fixed fusion method. The EL-APMC model is an adaptive fusion method called Power Mean Combiner (PMC). During the training process of the EL-APMC algorithm, the PMC is trained to match the statistics of the base classifier outputs of the EL-APMC model. In comparison to the standard ensemble-learning algorithm, for example, the ensemble bagged trees used a fixed fusion method called the majority-voting rule. Using a fixed fusion method in ensemble learning limited their predicting ability, especially in the case of limited dataset size.

To study the statistical significance of the results given in Table 5, we analyze the classification results (accuracy, recall, precision, and F1-score) in terms of a sample mean, sample standard deviation, and hypothesis tests. The purpose of the sample mean and the standard deviation is to evaluate the overall performance of machine learning algorithms. The results in Table 6 show the classification models achieved on average a good performance on recall (94.44%) and F1-score (91.22%) metrics. There is a high standard deviation in the classification results in terms of recall (0.0355) and precision (0.027) compared to the accuracy and F1 score metric. In other words, classifier models exhibit high variability in performance in terms of recall and precision compared to other metrics. The purpose of hypothesis tests is to make sure that the differences in the classification results have statistical significance or not. For this purpose, we use the one-sample Kolmogorov–Smirnov test. The null hypothesis states that the classification results in terms of accuracy, recall, precision, and F1-score come from a specific distribution versus the alternative hypothesis that the samples do not come from such a distribution at a 5% significance level. The *P*-value shown in Table 6 shows small values for all metrics i.e. less than 5% which means rejecting the null hypnosis and differences in the classification results have statistical significance.

**Table 6:** Statistical analysis for results of classification models.

Metrics	*P*-values	Mean	Standard deviation
Accuracy	1.4286e−12	0.8542	0.0194
Recall	4.5870e−13	0.9444	0.0355
Precision	1.3032e−12	0.8838	0.0270
F1-Score	2.4985e−13	0.9122	0.0118

In terms of estimating the complexity of the EL-APMC compared to other algorithms. We use the notation $O\left( n \right)$, where $n$ is the number of analyzing loops, recursive calls, and other control structures in the algorithm. In the training phase, EL-APMC used two main steps; training $N$ base classifiers and running a surrogate algorithm. In comparison to other machine learning algorithms used in this work, we can estimate the time complexity of the EL-APMC as $O\left( {N + M} \right)$. Where N is the linear time required to train $N$ base classifiers and $M$ is the number of time iterations needed by the surrogate algorithm to find the optimal $\alpha$. Another limitation of the EL-APMC is the fusion method used which offers limited search space. One possible solution is to use generalized *f*-mean which is considered as a general case of power mean combiner. Implementing generalized *f*-mean is expected to add more complexity since the searching space for the optional fusion method is expanded compared to the searching space of the power mean combiner.

## Conclusion and future work

There is a noticed lack of data availability for patients with brain tumors that is resulting in small data size. Classification of small-size datasets faces many challenges such as overfitting or underfitting problems that put limits on the ability of machine learning algorithms for classification. In this work, we applied an automated machine-learning classification system for glioma grades based on a novel MRI feature extraction method. We used an effective ensemble learning method called EL-APMC and evaluated its ability to classify a limited-size MRI dataset. We compare the performance of EL-APMC against 21 machine-learning methods that represent state-of-the-art classification models. Results show the EL-APMC algorithm outperforms the al1 classification models in terms of accuracy and F1-score metric. This score is considered crucial in the case of an imbalanced dataset when the number of samples in one class overwhelms the number of samples in another class. We believe that the EL-APMC are effective classification method in case of small and imbalanced datasets. The next step in this research is to employ the generalized f-mean, which is seen as a broader form of the power mean combiner. Broadening the scope of searching for an optimal fusion method is expected to enhance classification outcomes.

The proposed machine-learning algorithm based on the novel MRI feature extraction offers significant aid to assist clinicians in clinical diagnosis and may further reduce efforts and unnecessary invasive procedures like biopsies through the confirmation process for the malignancy grade of a brain tumor. In addition, the proposed algorithm can be utilized in any application that is related to the fusion of multi-source information.

## Data Availability

The dataset used in this study is available at https://www.smir.ch/BRATS/Start2015.
